# User-Friendly Chatbot to Mitigate the Psychological Stress of Older Adults During the COVID-19 Pandemic: Development and Usability Study

**DOI:** 10.2196/49462

**Published:** 2024-03-13

**Authors:** Ya-Hsin Chou, Chemin Lin, Shwu-Hua Lee, Yen-Fen Lee, Li-Chen Cheng

**Affiliations:** 1 Department of Psychiatry Taoyuan Chang Gung Memorial Hospital Taoyuan County Taiwan; 2 Department of Psychiatry Keelung Chang Gung Memorial Hospital Keelung City Taiwan; 3 College of Medicine Chang Gung University Taoyuan County Taiwan; 4 Community Medicine Research Center Chang Gung Memorial Hospital Keelung Taiwan; 5 Department of Psychiatry Linkou Chang Gung Memorial Hospital Taoyuan County Taiwan; 6 Department of Information and Finance Management National Taipei University of Technology Taipei Taiwan

**Keywords:** geriatric psychiatry, mental health, loneliness, chatbot, user experience, health promotion, older adults, technology-assisted interventions, pandemic, lonely, gerontology, elderly, develop, design, development, conversational agent, geriatric, geriatrics, psychiatry

## Abstract

**Background:**

To safeguard the most vulnerable individuals during the COVID-19 pandemic, numerous governments enforced measures such as stay-at-home orders, social distancing, and self-isolation. These social restrictions had a particularly negative effect on older adults, as they are more vulnerable and experience increased loneliness, which has various adverse effects, including increasing the risk of mental health problems and mortality. Chatbots can potentially reduce loneliness and provide companionship during a pandemic. However, existing chatbots do not cater to the specific needs of older adult populations.

**Objective:**

We aimed to develop a user-friendly chatbot tailored to the specific needs of older adults with anxiety or depressive disorders during the COVID-19 pandemic and to examine their perspectives on mental health chatbot use. The primary research objective was to investigate whether chatbots can mitigate the psychological stress of older adults during COVID-19.

**Methods:**

Participants were older adults belonging to two age groups (≥65 years and <65 years) from a psychiatric outpatient department who had been diagnosed with depressive or anxiety disorders by certified psychiatrists according to the *Diagnostic and Statistical Manual of Mental Disorders (Fifth Edition) (DSM-5*) criteria. The participants were required to use mobile phones, have internet access, and possess literacy skills. The chatbot’s content includes monitoring and tracking health data and providing health information. Participants had access to the chatbot for at least 4 weeks. Self-report questionnaires for loneliness, depression, and anxiety were administered before and after chatbot use. The participants also rated their attitudes toward the chatbot.

**Results:**

A total of 35 participants (mean age 65.21, SD 7.51 years) were enrolled in the trial, comprising 74% (n=26) female and 26% (n=9) male participants. The participants demonstrated a high utilization rate during the intervention, with over 82% engaging with the chatbot daily. Loneliness significantly improved in the older group ≥65 years. This group also responded positively to the chatbot, as evidenced by changes in University of California Los Angeles Loneliness Scale scores, suggesting that this demographic can derive benefits from chatbot interaction. Conversely, the younger group, <65 years, exhibited no significant changes in loneliness after the intervention. Both the older and younger age groups provided good scores in relation to chatbot design with respect to usability (mean scores of 6.33 and 6.05, respectively) and satisfaction (mean scores of 5.33 and 5.15, respectively), rated on a 7-point Likert scale.

**Conclusions:**

The chatbot interface was found to be user-friendly and demonstrated promising results among participants 65 years and older who were receiving care at psychiatric outpatient clinics and experiencing relatively stable symptoms of depression and anxiety. The chatbot not only provided caring companionship but also showed the potential to alleviate loneliness during the challenging circumstances of a pandemic.

## Introduction

As COVID-19 spread worldwide, many governments implemented stay-at-home, social distancing, and self-isolation measures to protect the most vulnerable people, including older adults, for whom deteriorating physical health could result in both physical and mental challenges [1,2]. As one of the most vulnerable groups to experience negative outcomes of COVID-19, older adults were instructed to self-quarantine and isolate themselves from others to mitigate the risk of infection. Consequently, they missed out on their standard interactions with others and spent substantial amounts of time in isolation. Limited contact with other people can lead to loss of social support, which is especially important for older adults [1,2]. Indeed, older adults who experienced social isolation during the pandemic have been negatively impacted by feelings of increased loneliness and reduced quality of life. Furthermore, the risks faced by individuals living alone, including older adults, are particularly pronounced with more challenging economic conditions [3,4]. Loneliness is defined as a subjective feeling manifesting as uneasiness and unhappiness linked to a lack of connection or inclusiveness with others, whereas social isolation describes the objective state of an individual’s social environment and interactional patterns [5]. Growing evidence shows that “loneliness” is closely related to physical and mental health in older adults; stronger feelings of loneliness can lead to the development of unhealthy lifestyle habits (eg, smoking and drinking) and a reduction in engagement with health-positive behaviors (eg, exercise and a nutritious diet) [6,7]. Loneliness is also associated with decreased sleep and sleep efficiency and poor subjective sleep quality in middle-aged and older adults, along with correlations to cognitive dysfunction, disability, cardiovascular disease, increased mortality, and even suicide [8-10]. Loneliness is also associated with negative emotion processing in the brain [11] and is a significant risk factor for anxiety and depression in older adults [12,13].

The prevalence of anxiety disorders and depression among older adults is high. Approximately 2% of adults older than 55 years are diagnosed with major depression, and 10%-15% of older adults have depressive symptoms [14]. Anxiety disorders are the most common psychiatric diseases, with a lifetime prevalence of up to 30%. Treatment of depressive and anxiety disorders in older adults is difficult and common challenges include poor drug efficacy, adverse drug reactions, and common drug interactions caused by polypharmacy. Coping with loneliness among older adults has potential benefits for depression and anxiety, as loneliness can affect older people’s physical and mental health, triggering psychological vulnerability, causing brain alterations beyond those associated with normal aging, and reducing intellectual or psychological flexibility caused by discrete and chronic stress [15]. Given the serious impact of social isolation and loneliness on older adults’ mental and physical health, researchers must investigate ways to mitigate these adverse health outcomes. As an assistive technology, chatbots and conversational agents may provide companionship and reduce the loneliness experienced because of the social restrictions imposed during a pandemic [7].

In recent years, there have been breakthroughs in the application of deep-learning techniques for natural language processing, allowing a variety of applications to be used in medical communication, disease detection, and treatment. For instance, chatbots have been developed to assist patients with autism and to provide social-skill training for specific fields such as mental health [16]. There is also a publicly listed application that provides cognitive behavioral therapy to improve anxiety and depression symptoms. Chatbots are also used for veterans as a screening tool for posttraumatic stress syndrome, and users of such applications have compared its use to interacting with real people, highlighting the chatbot’s ability to more freely and accurately reflect their own symptoms [17]. Additionally, chatbots used for mental illness discharge preparation can offer time-saving benefits for nurses and allow users to repeat questions without time pressure [18]. Upon reviewing previous research, it is evident that most chatbot users are relatively young, with limited availability of chatbots specifically developed for older adults in psychiatric clinics. While an overwhelming number of mental health apps are available to the public, these apps seldom focus on the needs of the older adult population. Furthermore, older adults can be apprehensive about using apps containing too many complex functions. This situation highlights the need to design and develop an older adult–user-friendly chatbot geared toward the specific interests of this population, while promoting their physical and mental health.

To address this gap, this study investigated the perspectives of geriatric psychiatric outpatient users of a mental health chatbot during the COVID-19 pandemic and sought to develop a chatbot for older adult users with anxiety and depressive disorders. This chatbot aims to offer companionship and promote self-awareness regarding emotions, sleep patterns, and physical activity to improve overall health. Therefore, our primary research objective was to examine whether chatbots can mitigate the psychological stress that older adults experienced during COVID-19.

## Methods

### Ethical Considerations

Ethical approval was granted by the Institutional Review Board of Chang Gung Memorial Hospital (202001915B0). The participants were recruited from the psychiatric outpatient department of the hospital between September 2021 and March 2022. Informed consent was obtained from each participant before study initiation. Data were deidentified by assigning a unique identifier code to each participant; only the study moderator has access to the file linking the code to the personal information, which is stored in a locked cabinet. Participants were provided with a travel compensation of ~US $15 for completing the postintervention questionnaires; no other compensation was provided for participation in the study.

### Participants

Participants comprised two groups of individuals aged either 65 years and older or younger than 65 years who were diagnosed with depressive or anxiety disorders, including persistent depressive disorder, major depressive disorder, generalized anxiety disorder, panic disorder, or adjustment disorder with anxiety or depression. Certified psychiatrists made diagnoses according to *Diagnostic and Statistical Manual of Mental Disorders (Fifth Edition) (DSM-5*) criteria.

During the time of the study, none of the participants had experienced suicidal ideation or had an increase in medication 3 months prior to the beginning of the study. Participants included in the study were literate, active mobile phone users with internet access, who had used the chatbot regularly for at least 4 weeks. We excluded patients who were not literate, those with severe physical illness, and those diagnosed with a major neurocognitive illness (eg, schizophrenia, bipolar disorder, brain injury, substance abuse, stroke history, and dementia). During the case referral period, only one patient was excluded due to a diagnosis of bipolar disorder. All participants completed a questionnaire regarding their demographic characteristics.

### Health Promotion Chatbot

#### Chatbot Design

**Figure 1:**
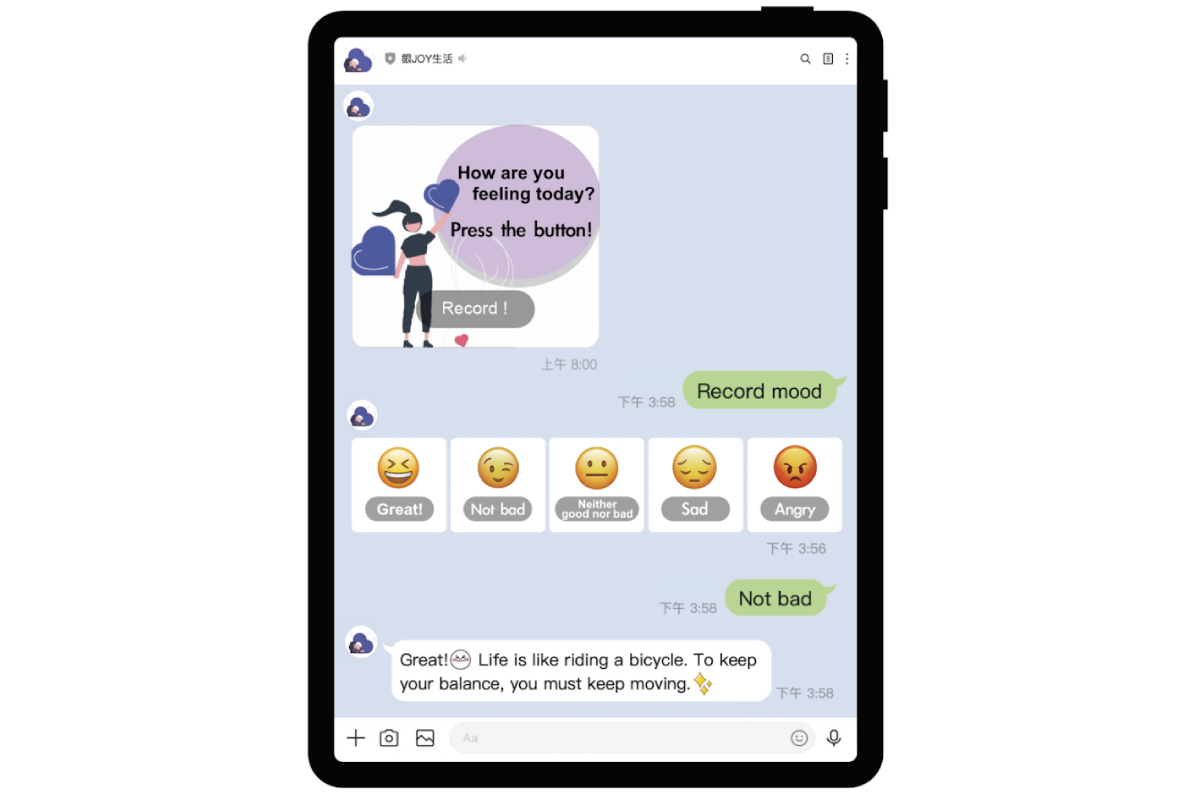
Mood tracking function of the Health Promotion chatbot.

**Figure 2:**
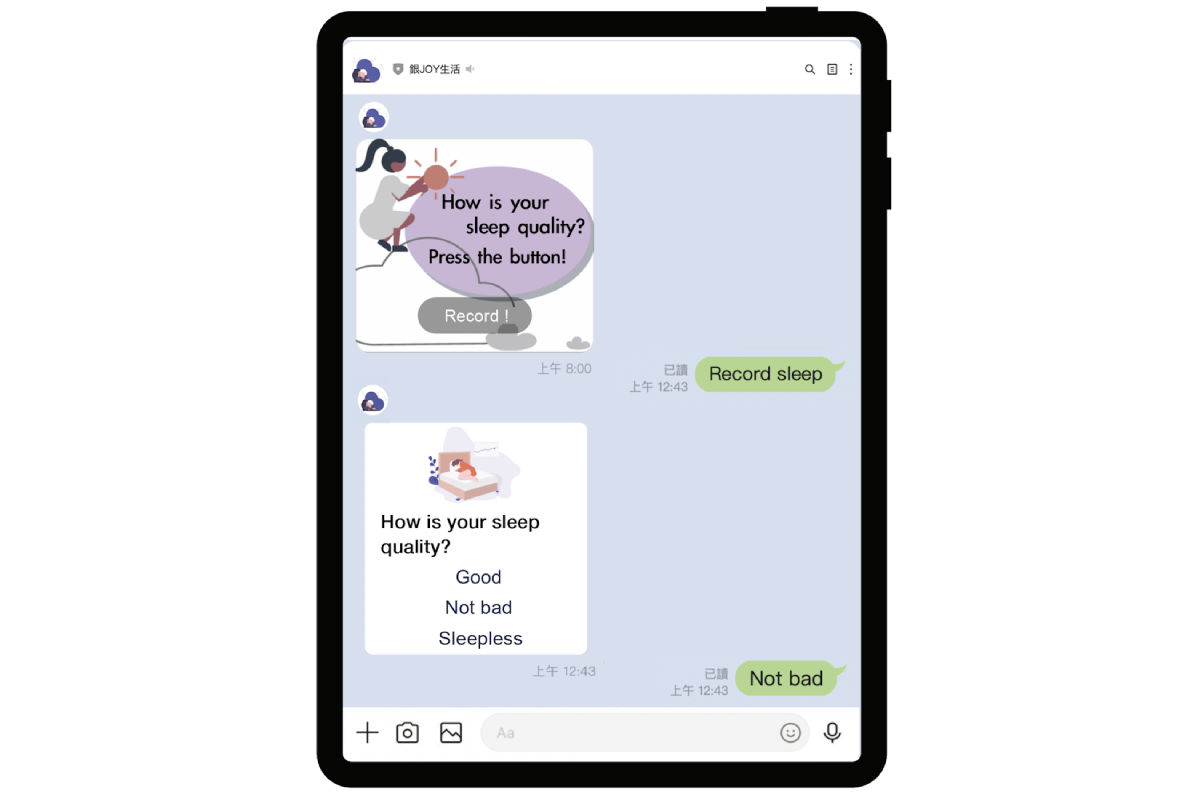
Sleep tracking function of the Health Promotion chatbot.

**Figure 3:**
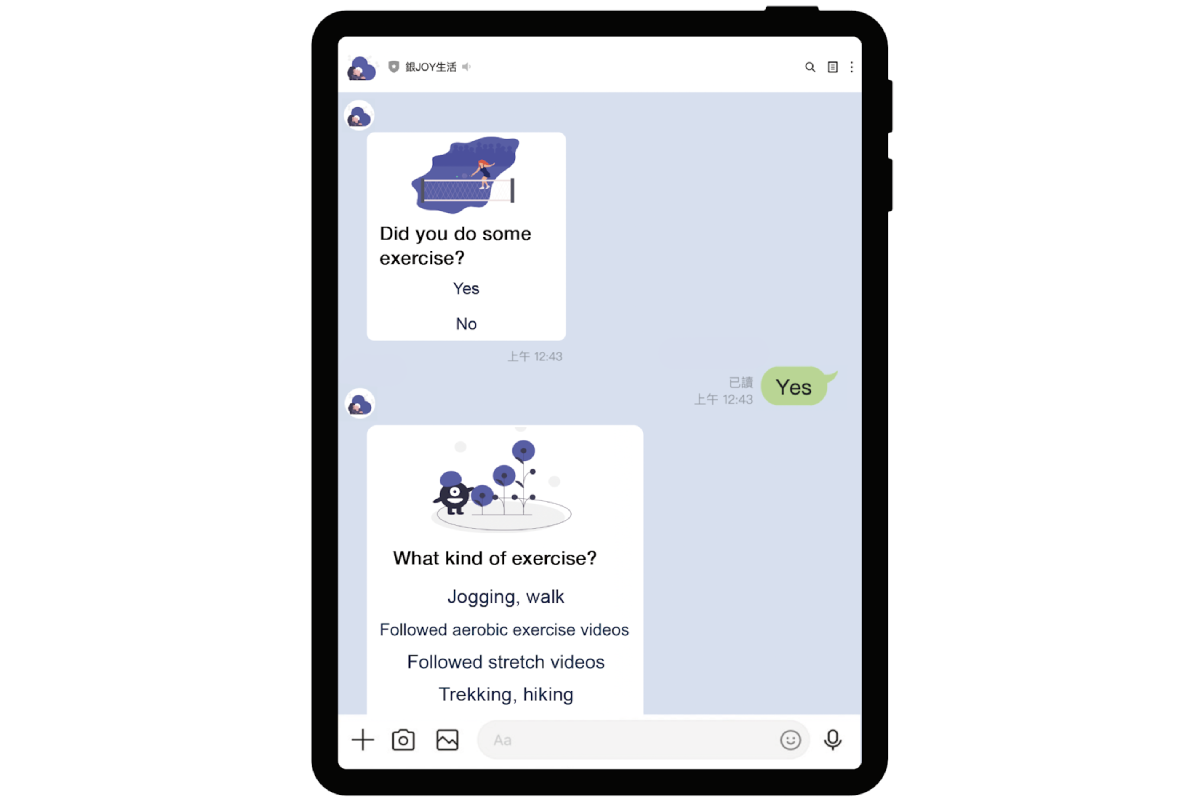
Daily activity tracking function of the Health Promotion chatbot.

**Figure 4:**
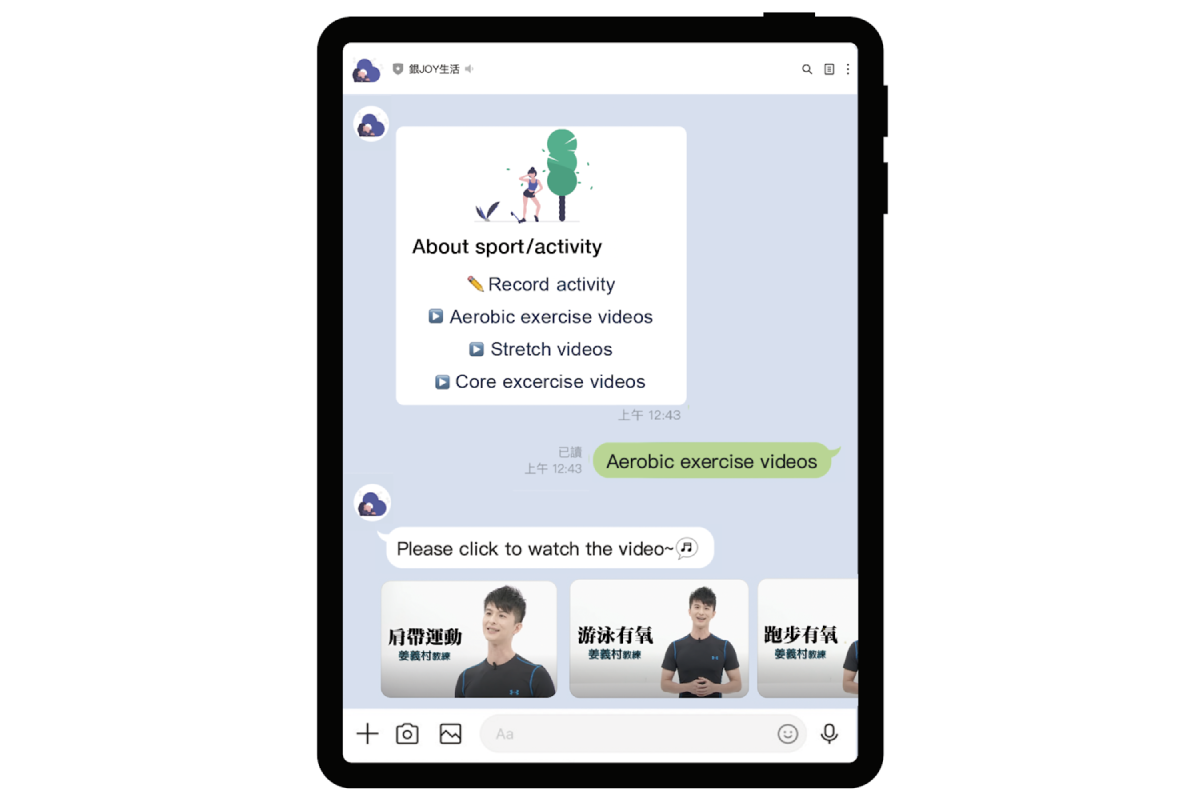
Health promotion education programs.

The LINE Official Account Manager was used to build the “Health Promotion” chatbot. LINE was used as it is currently the most popular chatbot for older adults in Taiwan. Previous research found that some older adults have difficulty keeping pencil-and-paper sleep diaries [19]. This difficulty can be ameliorated using mobile technologies; thus, we designed a user-friendly interface for older adults using buttons in the chatbot app. The proposed chatbot is multifunctional and includes health diary data collection and hygiene education functions. Figures 1-4 provide screenshots of the Health Promotion chatbot functions translated into English.

#### Monitoring and Recording Health Data

The Health Promotion chatbot sent daily messages to all participants and collected their health diary responses. The diaries tracked three key response areas, including mood, sleep, and daily activity. Figure 1 depicts the participants’ rating of their mood using five status terms: great, not bad, indifferent, sad, and angry. Sleep quality was rated as good, not bad, or sleepless (Figure 2). In addition to keeping an activity diary, the chatbot sent messages such as “Did you go out today?” or “Did you do some exercise? What kind of exercise?” (Figure 3). These messages helped guide older adult users to complete their activity information in the formatted “Message buttons.”

#### Providing Health Information and Advice

The main health promotion education program consisted of three topics: sleep hygiene education, food education, and exercise videos. The educational videos were provided to help the older adults practice good sleep hygiene and exercise habits. The sleep hygiene education video helped users practice “abdominal breathing” for relaxation. The exercise videos were recorded by a professional rehabilitator to guide older adults in performing different types of body movements. Overall, the app proved to be an effective tool to help the users perform training courses at home. The health promotion education programs are shown in Figure 4.

### Measures

#### Self-Assessments

Self-assessment scales for loneliness, depression, and anxiety were administered before and after chatbot use. Using a 7-point Likert scale, we also assessed individuals’ attitudes toward the chatbot post intervention.

Loneliness was measured using the University of California Los Angeles Loneliness Scale (UCLA-LS), which has demonstrated good reliability and validity for all ethnic groups as well as for older adults [20]. Participants rated themselves on a 1-4 scale for the 20 items, with a possible total score ranging from 20 to 80. As a one-dimensional measure, higher scores indicate greater loneliness.

Depression was measured using the Geriatric Depression Scale (GDS) 15-item short form; a cutoff score of 15 indicates clinical depression [21].

Anxiety was assessed using the Hospital Anxiety and Depression Scale anxiety subscale (HADS-A), which consists of 7 items. The depression subscale, also comprising 7 items, was not used in this study owing to the duplication of targets measured by the GDS [22].

#### Attitudes Toward the Chatbot

Based on the measure proposed by Bickmore et al [23], the questionnaire was modified to evaluate users’ attitudes toward the chatbot. The questionnaire covered four dimensions regarding satisfaction, usability, willingness to continue use, and adherence. All participants were asked to rate a series of attitude statements using a 7-point Likert rating scale, ranging from 1 (strongly disagree) to 7 (strongly agree), to assess their attitudes toward the chatbot’s features, such as “I was satisfied with the chatbot,” “It was easy to use the chatbot,” “I would like to continue using the chatbot,” “I will follow the chatbot’s recommendations,” and others [23-26].

#### Utilization Rate

The participant utilization rate was determined according to the frequency of interactions with the chatbot, specifically measured as the ratio of the number of interaction days to the total test days for each participant. This score encompasses the percentage of responses to inquiries regarding emotions, sleep quality, sport participation, and daily outings. For example, if a participant responded to emotional inquiries 15 times (with only one interaction per day counted) during a 30-day testing period, their emotional inquiry response rate would be 50%.

### Statistical Analysis

We compared the effectiveness of the chatbot between the age groups of ≥65 and <65 years. In Taiwan, individuals 65 years and older can enjoy old-age benefits (such as old-age pensions and bus discounts). Therefore, the division of participants into these two age groups allowed for a comparison of the effects of the chatbot on anxiety and depression in individuals who have already reached the age of eligibility for old-age benefits and those who have not. This distinction is important as it enables researchers to explore differences in psychological well-being between the two groups considering the impact of access to benefits and associated social support networks.

All statistical analyses were performed using SPSS for Windows 20.0 (IBM Corp). The Fisher exact test, Wilcoxon signed rank test, analysis of covariance, and Spearman rank correlations were applied as appropriate.

## Results

### Sample Characteristics

The age of the participants ranged from 55 to 82 years, with a mean age of 65.21 (SD 7.51) years; the study sample comprised 74% (n=26) female participants and 26% (n=9) male participants. There were no significant differences in the demographic variables between the groups, except that a greater percentage of participants in the older age group (≥65 years) were living alone (Table 1).

**Table 1:** Demographic characteristics of the participants in each group.

Characteristics		Entire cohort (N=35)	Aged ≥65 years (n=15)	Aged <65 years (n=20)	*P* value	
Age (years), mean (SD)		65.2 (7.4)	72.3 (4.9)	59.9 (3.2)	N/A	
**Gender, n (%)**						.24
	Female	26 (74)	13 (87)	13 (65)		
	Male	9 (26)	2 (13)	7 (35)		
**Marital status, n (%)**						.38
	Married	20 (57)	6 (40)	14 (70)		
	Single	5 (14)	3 (20)	2 (10)		
	Widowed	5 (14)	3 (20)	2 (10)		
	Divorced	5 (14)	3 (20)	2 (10)		
**Living situation, n (%)**						.02
	Living alone	8 (23)	7 (47)	1 (5)		
	Accompanied by family occasionally	4 (11)	1 (7)	3 (15)		
	Accompanied by family mostly	23 (66)	7 (47)	16 (80)		
**Current employment status, n (%)**						.12
	Employed	5 (14)	0 (0)	5 (25)		
	Unemployed/retired	25 (71)	12 (80)	13 (65)		
	Other	5 (14.3)	3 (20)	2 (10)		
**Systemic disease, n (%)**						.73
	Any systemic disease	19 (54)	9 (60)	10 (50)		
	Diabetes	5 (14)	1 (7)	4 (20)		
	Hypertension	13 (37)	8 (53)	5 (25)		
	Dyslipidemia	10 (29)	5 (33)	5 (25)		
**Psychiatric diagnosis, n (%)**						N/A^c^
	Major depressive disorder	9 (26)	4 (27)	5 (25)^b^		
	General anxiety disorder	6 (17)	2 (13)	4 (20)		
	Other anxiety disorder	20 (57)	9 (60)	11 (55)		
	Persistent depressive disorder	3 (9)	0 (0)	3 (15)		

^a^N/A: not applicable.

^b^One participant had comorbid major depressive and anxiety disorders.

^c^Statistical analysis was not applicable in this case since a single participant may have multiple psychiatric diagnoses.

### Attitude Toward the Chatbot

In the postintervention assessment, we collected the participants’ attitudes toward the chatbot using a 7-point Likert scale. The mean scores for satisfaction, usability, willingness to continue using, and adherence were 5.33, 6.33, 4.87, and 5.07, respectively, in the ≥65 years age group; the corresponding mean scores in the <65 years age group were 5.15, 6.05, 4.7, and 4.95, respectively. There were no group differences in satisfaction, usability, willingness to continue using, and adherence scores.

### Use and Response Rates

The use rate was determined by the frequency of a patient’s interaction with the chatbot, specifically measured as the ratio of interacting days to the total testing days for each participant. This included the rate of response to the inquiries for mood, sleep quality, and participation, as well as the “I went out today” inquiry.

There was no significant difference in the response rate between the two age groups. However, the response rate to the sport participation inquiry was less than 50%. Furthermore, during the 1-month intervention, the participants had a high use rate, with over 82% of the participants reporting being active daily (Table 2).

**Table 2:** Use and response rates in each group.

Use and response rates^a^	Aged ≥65 years (n=15)	Aged <65 years (n=20)
Chatbot use, %	82.55	83.38
Emotional inquiry response, %	84.19	80.88
Sleep quality response, %	82.55	83.37
Sport participation response, %	46.95	48.16
“I went out today” response, %	78.25	66.33

^a^Calculated as the ratio of interacting days to the total testing days for each participant.

### Self-Assessment Scales

The improvement in the UCLA-LS score post intervention was not significant in the <65 age group (*P*=.98) but was significant in the ≥65 age group (*P*=.006). HADS-A and GDS scores showed no significant improvements in either group (Table 3).

**Figure 5:**
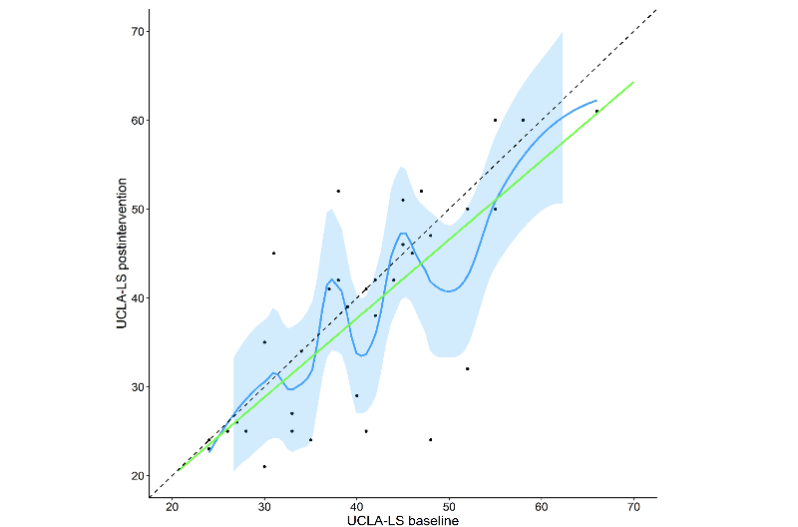
UCLA-LS scores post intervention versus baseline with LOESS fit and 95% confidence bands. UCLA-LS: University of California Los Angeles Loneliness Scale.

**Figure 6:**
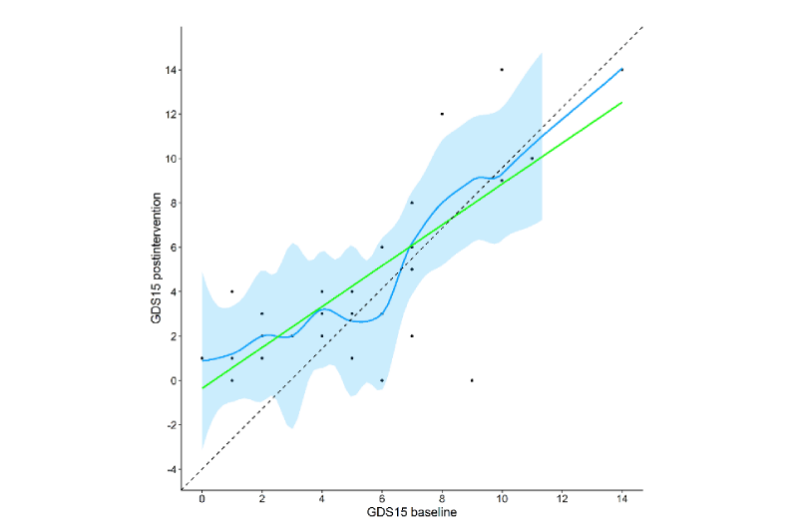
GDS-15 scores post intervention versus baseline with LOESS fit and 95% confidence bands. GDS-15: Geriatric Depression Scale 15-item short form.

**Figure 7:**
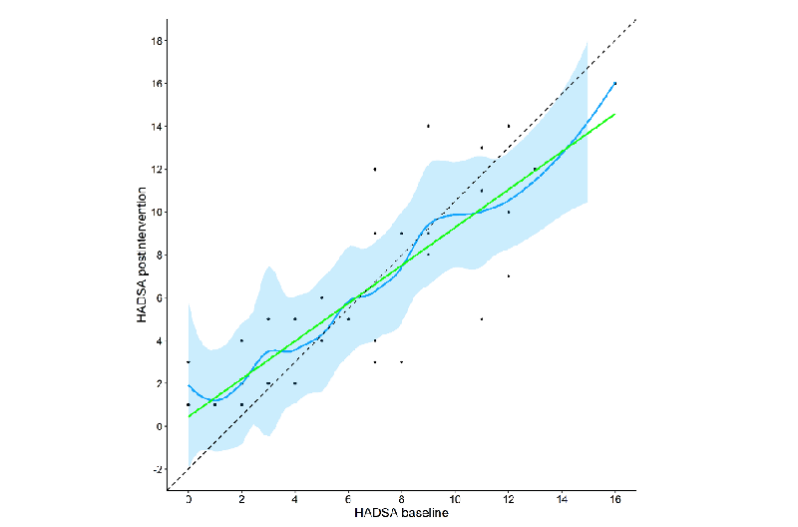
HADS-A scores post intervention versus baseline with LOESS fit and 95% confidence bands. HAD-A: Hospital Anxiety and Depression Scale anxiety subscale.

Figures 5-7 show the changed trends in the scales before and after the intervention. The slopes flattened after the intervention.

**Table 3:** Comparison of outcome measures at baseline and post intervention.

Outcome measures		Entire cohort (N=35), mean (SD)	Aged ≥65 years (n=15), mean (SD)	Aged <65 years (n=20), mean (SD)
**UCLA-LS^a^**				
	Baseline	40.4 (10.1)	38.9 (7.7)	41.5 (11.7)
	Postintervention	38.1 (12)	34.9 (9.2)	40.4 (13.5)
	*P* value	.12	.006	.98
**GDS^b^**				
	Baseline	4.9 (3.4)	3.6 (2.8)	5.8 (3.6)
	Postintervention	4.1 (4.1)	2.5 (1.8)	5.4 (4.9)
	*P* value	.07	.13	.30
**HADS-A^c^**				
	Baseline	6.3 (4.2)	4.3 (2.7)	7.8 (4.6)
	Postintervention	6.0 (4.5)	3.7 (2.1)	7.8 (5.0)
	*P* value	.61	.32	.92

^a^UCLA-LS: University of California Los Angeles Loneliness Scale.

^b^GDS: Geriatric Depression Scale.

^c^HADS-A: Hospital Anxiety and Depression Scale anxiety subscale.

## Discussion

### Principal Findings

#### Usability and User Satisfaction of the Health Promotion Chatbot Among Older Adults During the COVID-19 Pandemic

This study took place during the COVID-19 pandemic, which greatly impacted people’s daily lives and led to notable reductions in interpersonal interactions and outdoor activities [27]. The pandemic also substantially affected the economy, impacting employment and raising anxiety for many who experienced both the disease itself and the protocols set in place to counter the spread of the virus [28,29].

The results of this study indicate that both age groups, ≥65 and <65 years, were equally satisfied with the chatbot. Its design was rated highly for usability and user satisfaction by both groups. The ≥65 age group provided a mean satisfaction rating of 5.33 and a mean usability rating of 6.33, whereas the corresponding scores for the <65 age group were slightly lower at 5.15 and 6.05, respectively. These high satisfaction ratings reflect the chatbot’s effectiveness in catering to a wide range of users and their positive reception of the user-friendly interface.

The use rate among the older adults was remarkably positive. Previous studies have suggested that systems that are not user-friendly for older adults are often met with resistance and ineffectiveness [30]. However, this system, which was specifically designed for older adults, features a simple and user-friendly interface to target the barriers that older adult users might face in terms of digital literacy and technical assistance [31,32]. Our Health Promotion chatbot was designed to have concise functions, aiming to provide companionship and support even to those who are less familiar with technological products. Additionally, the use and response rates showed that age did not affect the willingness to use the chatbot, further suggesting that the content and interface of the chatbot are well-received and accessible for older adult users.

Therefore, the findings of this investigation on chatbot applicability in geriatric psychiatric outpatient clinics indicate that the chatbot interface is user-friendly and meets the desired criteria.

#### Chatbots Mitigate Loneliness in Older Adults

Some studies have explored the potential of assistive technology in addressing social isolation and loneliness among older adults. For example, one prospective cohort study reported that humanoid robots improve patient outcomes by providing companionship and entertainment in acute hospital settings and may be an acceptable outlet for socialization among older adults [33,34]. In a systematic review, Chen and Schulz [7] found that information and communication technology interventions can effectively reduce social isolation among older adults and potentially improve their quality of life by enhancing social support, promoting social engagement, and reducing feelings of loneliness and depression. However, a cross-sectional study also showed that problematic social media use was positively associated with perceived social isolation among older adults [35].

Four possible mechanisms by which information technology alleviates social isolation and loneliness have been proposed in the literature [7,36]: connection to the outside world, gaining social support, engaging in interesting activities, and boosting self-confidence. Boosting self-confidence was interpreted based on reports of older adults feeling younger and more equipped with new skills when taught how to use communication technologies. In our study, the chatbot greeted older adults daily and encouraged them to stay active. Some participants reported benefitting from the chatbot’s exercise videos; engaging with the chatbot and performing daily exercises made them feel rejuvenated and more purposeful.

Overall, our results suggest that the chatbot helped to reduce feelings of loneliness; the average UCLA-LS score of the total cohort at baseline was 40.4, which decreased to 38.1 post intervention. For the ≥65 age group, the average baseline score was 38.9, which decreased to 34.9 post intervention, representing a statistically significant difference (*P*=.006). However, the <65 age group had an average baseline score of 41.5, which remained stable post intervention with a nonsignificant difference (*P*=.98). Therefore, although the participants had decreased UCLA-LS scores post intervention on the whole, only the older participants (≥65 years) showed a significant improvement in loneliness.

Generally, the ≥65 age group responded well to the chatbot, as reflected in the UCLA-LS score changes, indicating that this age group can benefit from chatbots. Some potential reasons to explain the lack of an effect on loneliness for the <65 age group may be related to this group experiencing elevated stress in their daily lives [4,37] due to financial burdens and concerns about the future. Evidently, the effectiveness of chatbots in alleviating such stress is limited. Moreover, the <65 age group likely also had more opportunities for interpersonal interaction, even during the pandemic. Members of this group tend to not live alone and to have more avenues (eg, different types of social media) to access information. Consequently, the influence of chatbot interventions on their lives was comparatively less pronounced.

Our study demonstrates the positive impact of chatbot interventions on older adults enrolled in psychiatric outpatient departments and with relatively stable symptoms during the pandemic. Previous studies have mentioned the importance of using technology to reduce the impact of loneliness on older adults, particularly in the context of pandemic-related restrictions [2,38,39]. Although this study was conducted during a period when the pandemic was more severe, it offers valuable insights into older adult care. To enhance the generalizability and applicability of future trials, we recommend expanding the participant pool to include older adults outside of clinical settings.

### Limitations

This pilot study has some limitations. When assessing loneliness and depression, it is important to include in-depth interviews to understand users’ thoughts. However, due to the pandemic, interviews could not be conducted during the study period. We therefore aim to further enhance this aspect by including interviews in future research. In addition, given that the study was conducted during the COVID-19 pandemic, it was challenging to enroll participants, as many patients were unwilling to visit the hospital, resulting in a limited sample size. The improvement in loneliness may be due in part to the placebo effect. Additionally, the study sample was derived solely from the psychiatric outpatient department of a single medical center, potentially introducing bias in the referral process and limiting the generalizability of the findings to a broader population. Finally, the follow-up period was relatively short (within half a year); thus, determining the long-term effect of the intervention requires further research.

### Conclusions

The chatbot interface developed in this study was simple and easy to use. The results demonstrated that older adults in psychiatric outpatient clinics with relatively stable depressive and anxiety symptoms could benefit from the chatbot’s care, companionship, and potential reduction in loneliness, particularly during a pandemic. Simultaneously, the accessibility and user satisfaction of chatbots, which are crucial for promoting the health of older adults through using such apps, enabled the participants to easily access health information and advice, thereby enhancing their health awareness and quality of life.

Future research can explore how more personalized mental health support can be provided by expanding on the functionality of chatbots to effectively manage depression and anxiety among older adults. Simultaneously, there should be in-depth exploration of how chatbots can be integrated with traditional health care services to establish a more comprehensive medical support system. Qualitative investigations through individual or group interviews can add further insights into their use and adaptation.

## Abbreviations

DSM-5: Diagnostic and Statistical Manual of Mental Disorders (Fifth Edition)
GDS: Geriatric Depression Scale
HADS-A: Hospital Anxiety and Depression Scale anxiety subscale
UCLA-LS: University of California Los Angeles Loneliness Scale

## Data Availability

All data generated or analyzed during this study are included in the published article.

## References

[1] Gerst-Emerson K, Jayawardhana J (2015). Loneliness as a public health issue: the impact of loneliness on health care utilization among older adults. Am J Public Health.

[2] Armitage R, Nellums LB (2020). COVID-19 and the consequences of isolating the elderly. Lancet Public Health.

[3] Sutin AR, Luchetti M, Terracciano A (2020). Has loneliness increased during COVID-19? Comment on "Loneliness: A signature mental health concern in the era of COVID-19". Psychiatry Res.

[4] González-Sanguino C, Ausín B, Castellanos MÁ, Saiz J, López-Gómez A, Ugidos C, Muñoz M (2020). Mental health consequences during the initial stage of the 2020 Coronavirus pandemic (COVID-19) in Spain. Brain Behav Immun.

[5] Victor CR, Scambler SJ, Bowling A, Bond J (2005). The prevalence of, and risk factors for, loneliness in later life: a survey of older people in Great Britain. Ageing Soc.

[6] Ong AD, Uchino BN, Wethington E (2016). Loneliness and health in older adults: a mini-review and synthesis. Gerontology.

[7] Chen YR, Schulz PJ (2016). The effect of information communication technology interventions on reducing social isolation in the elderly: a systematic review. J Med Internet Res.

[8] Morley JE, Vellas B (2020). Editorial: COVID-19 and older adults. J Nutr Health Aging.

[9] Valtorta NK, Kanaan M, Gilbody S, Ronzi S, Hanratty B (2016). Loneliness and social isolation as risk factors for coronary heart disease and stroke: systematic review and meta-analysis of longitudinal observational studies. Heart.

[10] Rafnsson S, Orrell M, d'Orsi E, Hogervorst E, Steptoe A (2020). Loneliness, social integration, and incident dementia over 6 years: prospective findings from the English longitudinal study of ageing. J Gerontol B Psychol Sci Soc Sci.

[11] Wong NML, Liu H, Lin C, Huang C, Wai Y, Lee S, Lee TMC (2016). Loneliness in late-life depression: structural and functional connectivity during affective processing. Psychol Med.

[12] Cacioppo JT, Hughes ME, Waite LJ, Hawkley LC, Thisted RA (2006). Loneliness as a specific risk factor for depressive symptoms: cross-sectional and longitudinal analyses. Psychol Aging.

[13] Cacioppo JT, Hawkley LC, Thisted RA (2010). Perceived social isolation makes me sad: 5-year cross-lagged analyses of loneliness and depressive symptomatology in the Chicago Health, Aging, and Social Relations Study. Psychol Aging.

[14] Kok RM, Reynolds CF (2017). Management of depression in older adults: a review. JAMA.

[15] Fitten L (2015). Psychological frailty in the aging patient. Nestle Nutr Inst Workshop Ser.

[16] Abd-Alrazaq AA, Alajlani M, Alalwan AA, Bewick BM, Gardner P, Househ M (2019). An overview of the features of chatbots in mental health: a scoping review. Int J Med Inform.

[17] Lucas GM, Rizzo A, Gratch J, Scherer S, Stratou G, Boberg J, Morency L (2017). Reporting mental health symptoms: breaking down barriers to care with virtual human interviewers. Front Robot AI.

[18] Vaidyam AN, Wisniewski H, Halamka JD, Kashavan MS, Torous JB (2019). Chatbots and conversational agents in mental health: a review of the psychiatric landscape. Can J Psychiatry.

[19] Oh ES, Park KM, An SK, Namkoong K (2018). The effects of a brief intervention for insomnia on community dwelling older adults. Sleep Med Psychophysiol.

[20] Carson SH, Peterson JB, Higgins DM (2005). Reliability, validity, and factor structure of the creative achievement questionnaire. Creativ Res J.

[21] Shin C, Park MH, Lee S, Ko Y, Kim Y, Han K, Jeong H, Han C (2019). Usefulness of the 15-item geriatric depression scale (GDS-15) for classifying minor and major depressive disorders among community-dwelling elders. J Affect Disord.

[22] Brehaut E, Neupane D, Levis B, Wu Y, Sun Y, Krishnan A, He C, Bhandari PM, Negeri Z, Riehm KE, Rice DB, Azar M, Yan XW, Imran M, Chiovitti MJ, Saadat N, Cuijpers P, Ioannidis JPA, Markham S, Patten SB, Ziegelstein RC, Henry M, Ismail Z, Loiselle CG, Mitchell ND, Tonelli M, Boruff JT, Kloda LA, Beraldi A, Braeken APBM, Carter G, Clover K, Conroy RM, Cukor D, da Rocha E Silva CE, De Souza J, Downing MG, Feinstein A, Ferentinos PP, Fischer FH, Flint AJ, Fujimori M, Gallagher P, Goebel S, Jetté N, Julião M, Keller M, Kjærgaard M, Love AW, Löwe B, Martin-Santos R, Michopoulos I, Navines R, O'Rourke SJ, Öztürk A, Pintor L, Ponsford JL, Rooney AG, Sánchez-González R, Schwarzbold ML, Sharpe M, Simard S, Singer S, Stone J, Tung KY, Turner A, Walker J, Walterfang M, White J, Benedetti A, Thombs BD (2020). Depression prevalence using the HADS-D compared to SCID major depression classification: an individual participant data meta-analysis. J Psychosom Res.

[23] Bickmore TW, Pfeifer LM, Byron D, Forsythe S, Henault LE, Jack BW, Silliman R, Paasche-Orlow MK (2010). Usability of conversational agents by patients with inadequate health literacy: evidence from two clinical trials. J Health Commun.

[24] Abd-Alrazaq A, Safi Z, Alajlani M, Warren J, Househ M, Denecke K (2020). Technical metrics used to evaluate health care chatbots: scoping review. J Med Internet Res.

[25] Griffin AC, Khairat S, Bailey SC, Chung AE (2023). A chatbot for hypertension self-management support: user-centered design, development, and usability testing. JAMIA Open.

[26] Milne-Ives M, de Cock C, Lim E, Shehadeh MH, de Pennington N, Mole G, Normando E, Meinert E (2020). The effectiveness of artificial intelligence conversational agents in health care: systematic review. J Med Internet Res.

[27] O'Sullivan R, Burns A, Leavey G, Leroi I, Burholt V, Lubben J, Holt-Lunstad J, Victor C, Lawlor B, Vilar-Compte M, Perissinotto CM, Tully MA, Sullivan MP, Rosato M, Power JM, Tiilikainen E, Prohaska TR (2021). Impact of the COVID-19 pandemic on loneliness and social isolation: a multi-country study. Int J Environ Res Public Health.

[28] Hossain MM, Tasnim S, Sultana A, Faizah F, Mazumder H, Zou L, McKyer ELJ, Ahmed HU, Ma P (2020). Epidemiology of mental health problems in COVID-19: a review. F1000Res.

[29] Gloster AT, Lamnisos D, Lubenko J, Presti G, Squatrito V, Constantinou M, Nicolaou C, Papacostas S, Aydın G, Chong YY, Chien WT, Cheng HY, Ruiz FJ, Garcia-Martin MB, Obando-Posada DP, Segura-Vargas MA, Vasiliou VS, McHugh L, Höfer S, Baban A, Dias Neto D, Nunes da Silva A, Monestès JL, Alvarez-Galvez J, Paez-Blarrina M, Montesinos F, Valdivia-Salas S, Ori D, Kleszcz B, Lappalainen R, Ivanović I, Gosar D, Dionne F, Merwin RM, Kassianos AP, Karekla M (2020). Impact of COVID-19 pandemic on mental health: an international study. PLoS One.

[30] Iancu I, Iancu B (2020). Designing mobile technology for elderly. A theoretical overview. Technol Forecast Soc Change.

[31] Frishammar J, Essén A, Bergström F, Ekman T (2023). Digital health platforms for the elderly? Key adoption and usage barriers and ways to address them. Technol Forecast Soc Change.

[32] Yusif S, Soar J, Hafeez-Baig A (2016). Older people, assistive technologies, and the barriers to adoption: a systematic review. Int J Med Inform.

[33] Sarabia M, Young N, Canavan K, Edginton T, Demiris Y, Vizcaychipi MP (2018). Assistive robotic technology to combat social isolation in acute hospital settings. Int J of Soc Robotics.

[34] Chung K, Kim S, Lee E, Park JY (2020). Mobile app use for insomnia self-management in urban community-dwelling older Korean adults: retrospective intervention study. JMIR Mhealth Uhealth.

[35] Meshi D, Cotten S, Bender A (2020). Problematic social media use and perceived social isolation in older adults: a cross-sectional study. Gerontology.

[36] Grist R, Porter J, Stallard P (2017). Mental health mobile apps for preadolescents and adolescents: a systematic review. J Med Internet Res.

[37] Nwachukwu I, Nkire N, Shalaby R, Hrabok M, Vuong W, Gusnowski A, Surood S, Urichuk L, Greenshaw AJ, Agyapong VI (2020). COVID-19 pandemic: age-related differences in measures of stress, anxiety and depression in Canada. Int J Environ Res Public Health.

[38] Rodney T, Josiah N, Baptiste D (2021). Loneliness in the time of COVID-19: impact on older adults. J Adv Nurs.

[39] Jutai JW, Tuazon JR (2022). The role of assistive technology in addressing social isolation, loneliness and health inequities among older adults during the COVID-19 pandemic. Disabil Rehabil Assist Technol.

